# New Insight Into Male Infertility: Deviation From Basic Tenets of Life

**DOI:** 10.1155/omcl/4433444

**Published:** 2026-08-30

**Authors:** Mohammad Hossein Nasr-Esfahani, Nushin Naderi, Marziyeh Tavalaee, Mazdak Razi

**Affiliations:** ^1^ Department of Animal Biotechnology, Reproductive Biomedicine Research Center, Royan Institute for Biotechnology, ACECR, Isfahan, Iran, acecr.ac.ir; ^2^ Pooyesh and Rooyesh Fertility Center, Isfahan, Iran; ^3^ Division of Comparative Histology and Embryology, Department of Basic Science, Faculty of Veterinary Medicine, Urmia University, Urmia, Iran, urmia.ac.ir

**Keywords:** endocrine disruptors, fructose metabolism, male infertility, obesity, oxidative stress

## Abstract

Male reproductive health faces mounting concern as global sperm concentration (SC) has declined markedly, with evidence linking this trend to environmental and lifestyle factors rather than natural variability. In parallel, rising rates of obesity, diabetes, and hypertension point to shared mechanisms with infertility, particularly those rooted in diet. High intake of processed sugars, especially high‐fructose corn syrup (HFCS), contributes to metabolic dysfunction, oxidative stress, hormonal imbalance, and inflammation, all of which can impair sperm function and disrupt the hypothalamic–pituitary–gonadal (HPG) axis. Unlike glucose, fructose undergoes hepatic metabolism, fostering insulin resistance, fat accumulation, and nonalcoholic fatty liver disease (NAFLD), further aggravating reproductive risks. In this review, four models of obesity, the energy balance model (EBM), carbohydrate‐insulin model (CIM), obesogen model, and OBS/REDOX model, are applied to explain how excess fructose and obesogenic exposures drive metabolic dysfunction, oxidative stress, and endocrine disruption. Collectively, these processes reduce testosterone levels, impair spermatogenesis, compromise sperm quality, and may even induce epigenetic alterations transmissible to future generations. With modern diets increasingly dominated by HFCS and ultra‐processed foods (UPFs), understanding fructose’s role in reproductive decline is crucial for developing effective interventions. This review underscores the need for further research to clarify how dietary fructose and environmental obesogens jointly shape metabolic and reproductive health, offering insights into public health strategies aimed at protecting male fertility in the face of modern lifestyle challenges.

## 1. Introduction

In 2017, Levine et al. [1] published a landmark meta‐analysis reporting a nearly 50% decline in sperm count among Western men between 1973 and 2011, a trend potentially associated with adverse social and environmental factors affecting male reproductive health. Despite early skepticism, this reported decline has received considerable attention in andrology, and some researchers have introduced the term sperm count biovariability (SCB) to account for natural fluctuations in sperm parameters across populations [2]. Nonetheless, sperm count decline (SCD) has been described as a “canary in the coal mine” for male health, both as a biomarker of current well‐being and a predictor of future health risks [1]. However, critics argue that spermatogenesis is influenced by a multitude of factors, making sperm count an insufficient standalone metric for assessing men’s health [2].

Alongside several idiopathic and environmental causes of infertility and in parallel with the ongoing decline in sperm counts [3], the global prevalence of metabolic diseases [4]—including obesity [5], diabetes [6], dyslipidemia [7], insulin resistance [8], hypertension [9], hyperuricemia [10], and nonalcoholic fatty liver disease (NAFLD) [11]—has surged. Among these conditions, obesity has seen one of the most dramatic increases, with 2.5 billion adults classified as overweight in 2022, including over 890 million categorized as obese. The prevalence of overweight varied significantly by region, ranging from 31% in South‐East Asia and Africa to 67% in the Americas. Globally, 16% of adults were considered obese that year, marking a doubling in obesity rates from 1990 to 2022 [12].

This sharp rise in metabolic disorders cannot be attributed to genetics alone; rather, it reflects the profound influence of modifiable lifestyle factors, particularly diet and physical activity. In recent decades, dietary patterns have shifted dramatically, with increasing consumption of sugary foods and beverages replacing more nutrient‐dense options [13]. While fruits, a natural source of dietary sugar, provide health benefits due to their fiber and phytochemical content [14], added sugars—particularly high‐fructose corn syrup (HFCS), which contains the monosaccharides glucose and fructose—may exert distinct metabolic effects [15, 16]. The availability and consumption of HFCS have increased substantially because of industrial advances and agricultural subsidies [17]. Between 1970 and 1990, HFCS consumption in the United States alone surged by 1000%, far outpacing the intake of other food groups [18].

These dietary shifts may have consequences extending beyond traditional metabolic disorders. Emerging evidence suggests that poor dietary habits and metabolic dysregulation may influence reproductive health, immune function, gut microbiota composition, neuroendocrine signaling, and epigenetic programing [19, 20]. These systemic effects underscore the complex interplay between metabolic health and overall physiological function, with implications that may span generations [21, 22]. Although metabolic syndrome (MetS) is widely acknowledged as a risk factor for male infertility, the specific contribution of high fructose intake, a potential contributor to metabolic alterations, remains largely underexplored in the context of reproductive dysfunction.

More specifically, emerging evidence suggests that high fructose consumption may directly disrupt physiological processes beyond energy metabolism as excessive intake has been linked to hormonal imbalances, increased oxidative stress, and inflammation [23]—factors that can adversely affect reproductive function [24, 25]. These findings suggest a possible association between high fructose intake and male reproductive impairments, supporting further investigation into whether dietary patterns that promote MetS may also contribute directly to male infertility.

This article provides an in‐depth analysis of current research on the relationships among fructose metabolism, metabolic dysfunction, and male infertility. It explores the shared pathophysiological mechanisms linking metabolic disorders to impaired male reproductive function, with a particular focus on fructose as a major dietary contributor to obesogenic metabolic pathways. By examining the molecular and cellular processes that connect metabolic disturbances to impaired reproductive function, the article highlights how dietary factors—especially fructose—can amplify these reproductive challenges. Together, the evidence reviewed here provides a foundation for future studies in this important yet underexplored field.

## 2. Sperm Count and Male Infertility

Male infertility is a multifaceted issue influenced by both biological and social factors and cannot be assessed solely using sperm count metrics [26, 27]. While azoospermia, characterized by a complete absence of sperm, unequivocally results in infertility, evidence suggests that men with low sperm counts may still achieve conception, whereas those with higher sperm counts are not necessarily fertile [28, 29].

Guzick et al.[27] reported that sperm concentrations (SCs) in the subfertile range (less than 13.5 million/mL) do not necessarily exclude normal fertility. Furthermore, the 2021 WHO reference values for semen parameters, established from studies of fertile men [30], cannot accurately distinguish between fertile and infertile men at the individual level; many men below the 5th percentile may still be fertile. Global studies have similarly shown that those men with sperm counts below the WHO reference values can still achieve fertility [31–33].

Since the mid‐1970s, concerns about diminishing human reproductive health have been prevalent in industrialized nations. This concern has arisen partly from revisions of minimum semen quality standards and observations of declining SCs over time. The discourse on male infertility gained further momentum following the meta‐analysis by Levine et al. [1], which reported a more than 50% decline in SC and total sperm count (TSC) among Western men between 1973 and 2011. These findings formed the basis of the SCD hypothesis, which proposes that SC and TSC have been progressively decreasing since the early 1970s. Although the decline was initially observed primarily in Western populations, an expanded analysis based on studies published between 1981 and 2019 [34] later extended the dataset to include South/Central America, Asia, and Africa, where a significant decrease in SC and TSC was also detected for the first time. Moreover, post‐2000 data suggested that the rate of decline had accelerated globally. Because sperm count has been proposed as an indicator of male health across the lifespan, these observations have raised concerns regarding both male reproductive health and broader public health implications. Proposed contributing factors include exposure to endocrine‐disrupting chemicals (EDCs) and other environmental pollutants associated with industrialization as well as contemporary lifestyle changes.

Amid these concerns, the SCB hypothesis proposed by Boulicault et al.[2] offers an alternative interpretation of sperm count trends. It underscores that sperm counts vary widely and that much of this variation may be non‐pathological and species‐typical. Moreover, even sperm counts exceeding the WHO threshold may not reflect optimal fertility potential because semen parameters can vary considerably across individuals, ecologies, and time periods due to external influences. Although the SCB hypothesis does not disregard the possible effects of declining sperm counts on population fertility, it posits that the observed trends may represent adaptive variations rather than a straightforward decline in male reproductive health. While the SCB hypothesis highlights the importance of biological variability, the persistence and geographic consistency of the observed decline suggest that broader environmental and lifestyle factors may be contributing to these trends.

Levine et al.[34] argued that the observed decline in sperm count is unlikely to be the result of random “sperm variability,” as meta‐analyses demonstrated a consistent downward trajectory rather than the balanced pattern of increases and decreases that would be expected from random fluctuation. Sperm count, while not a perfect indicator of fertility, is strongly correlated with fertility potential, as evidenced by Guzick et al. [27]. The relationship between SC and time to conception is nonlinear; beyond a threshold of 40–50 million/mL, an increase in SC does not significantly enhance the likelihood of conception. Conversely, below this threshold, the probability of conception declines sharply as SC decreases [35]. On a population scale, the decline in average SC from 104 to 49 million/mL, as reported in that analysis, suggests a notable rise in the proportion of men experiencing delayed conception. Accordingly, SC remains one of the most consistent and reliable metrics for comparing fertility trends within and across populations over time [34].

In brief, male infertility is a complex and multifactorial condition that cannot be fully explained by sperm count metrics alone. However, at the population level, persistent declines in mean sperm counts documented across multiple continents [1, 34] cannot be readily dismissed as benign biological variability. Although sperm count alone does not determine individual reproductive outcomes, its strong association with time to conception and its reproducibility in epidemiological studies make it an important surveillance marker of male reproductive health.

Therefore, the ongoing debate between the SCD and SCB hypotheses should not obscure the broader public health implications. Sustained downward shifts in population sperm parameters warrant continued monitoring, deeper mechanistic investigation, and careful evaluation of environmental and lifestyle determinants that may contribute to the observed reductions in sperm quality and reproductive health. In this context, examining dietary exposures such as HFCS in relation to sperm count trends [24, 36] may provide valuable insights into contemporary influences on male fertility. Overall, integrating metabolic and metabolomic perspectives may help clarify the biological pathways linking modern environmental and dietary exposures to alterations in male reproductive function.

## 3. Fructose as a Key Driver of Metabolic Disease

**Figure 1:**
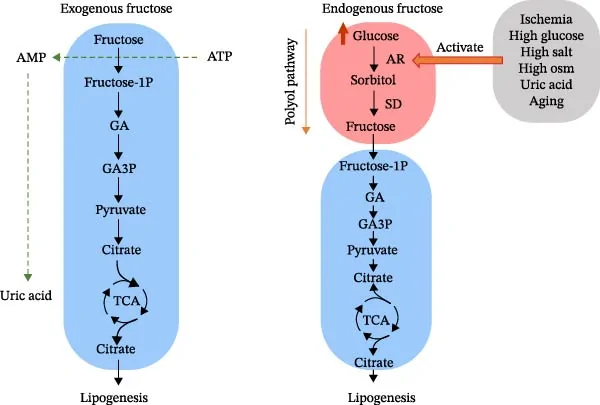
A diagram illustrating the body’s endogenous production of fructose through the polyol pathway in humans. The polyol pathway involves two enzymes: aldose reductase, which converts glucose to sorbitol, and sorbitol dehydrogenase, which further converts it to fructose. Although fructose can be reverted to glucose, its metabolism can increase triglycerides and uric acid, contributing to metabolic syndrome. Six mechanisms have been identified that enhance the expression of aldose reductase and the polyol pathway: ischemia, high glucose levels, high salt intake, high osmolality, uric acid, and aging.

The core principle of evolution centers on the availability of adequate food, water, and oxygen, fundamental necessities that, although often taken for granted in modern civilization, remain vital for the survival of many animals and were crucial for our distant ancestors [37, 38]. In this regard, Johnson et al. [39] proposed that fructose is a key mediator of an evolutionarily conserved survival pathway that supports adaptation to adverse environmental conditions. This pathway promotes energy and water storage and may also facilitate adaptation to reduced oxygen availability. Fructose is a simple sugar found mainly in fruits and honey. In a typical Western diet, it primarily comes from table sugar (sucrose) and HFCS, both of which are widely consumed sources of added fructose [40]. Currently, sugars in foods and beverages contribute ~15% of total energy intake, with some groups consuming more than 20% [41]. In addition to exogenous fructose, fructose can be produced in human cells through the polyol pathway (Figure 1), where aldose reductase (AR) converts glucose to sorbitol, which is then metabolized to fructose by sorbitol dehydrogenase (SDH). AR activity can be increased by high glucose levels (a common feature of diabetes), high‐salt diets, heat, tissue hypoxia, oxidative stress, fructose, and uric acid [39, 42].

Fructose, whether derived from exogenous sources or generated endogenously through the activation of the polyol pathway, plays a key role in shifting metabolism toward the synthesis of fat and glycogen, processes that facilitate energy storage and water retention during times of physiological stress or limited resources. In a natural setting, this metabolic adaptation is typically regulated by seasonal cycles [39]. For instance, increased fructose availability during summer is naturally followed by fall and winter, periods characterized by lower food availability and increased energy demands. This cyclical pattern may prevent chronic overstimulation of these metabolic pathways and thereby help maintain homeostasis [39]. Conversely, modern environments characterized by continuous access to high‐fructose foods—particularly HFCS and sugar‐sweetened beverages (SSBs), which are major sources of added sugars and fructose for both children and adults—may disrupt this evolutionary balance and contribute to metabolic dysfunction [43].

Overall, excessive fructose exposure, whether resulting from the dietary intake of HFCS and SSBs or increased endogenous production, may disrupt metabolic regulation and lead to chronic metabolic stress. While fructose once served as a vital energy source during times of scarcity, its constant presence in the modern diet may override natural regulatory mechanisms, thereby promoting sustained fat and glycogen synthesis. This persistent metabolic stimulation has been increasingly linked to systemic dysfunctions, including those that affect male reproductive health [44–47]. Accordingly, understanding the adverse effects of excessive fructose exposure requires consideration of how glucose and fructose, despite providing similar amounts of energy, exert distinct metabolic effects.

## 4. Glucose and Fructose Exert Distinct Metabolic Effects

During glycolysis, glucose is phosphorylated by glucokinase to form glucose‐6‐phosphate. This compound is subsequently phosphorylated by phosphofructokinase‐1 (PFK‐1) to produce fructose‐1,6‐bisphosphate (F1,6‐BP). F1,6‐BP is then cleaved by aldolase into two three‐carbon molecules: glyceraldehyde 3‐phosphate (GA3P) and dihydroxyacetone phosphate (DHAP) [48, 49]. Fructolysis begins with the phosphorylation of fructose by the enzyme fructokinase (FK), forming fructose‐1‐phosphate (F‐1‐P). Aldolase then cleaves F‐1‐P into two products: DHAP and glyceraldehyde (GA). These molecules can be converted into GA3P, which in turn is converted to pyruvate. This pathway ultimately leads to the generation of acety‐CoA. Acetyl‐CoA is crucial for the tricarboxylic acid cycle (TCA cycle, also known as the Krebs cycle or the citric acid cycle), where it contributes to ATP production or can be directed toward the synthesis of fatty acids and triglycerides (TGs) [39, 50, 51].

**Figure 2:**
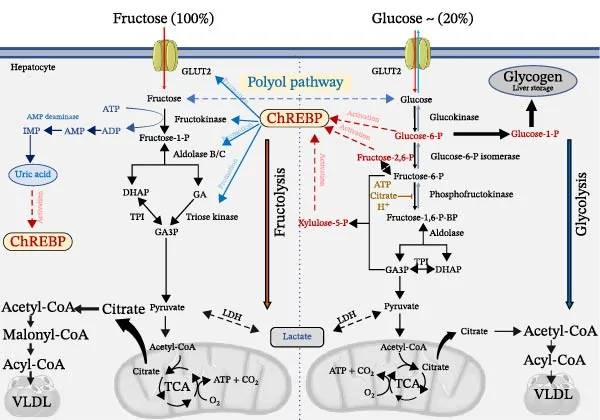
The hepatocyte metabolism of fructose and glucose. Fructose and glucose, despite both utilizing GLUT‐2 for uptake, follow distinct metabolic pathways. Fructose is phosphorylated at position 1 by hexokinase, leading to aldolase cleavage, which produces precursor metabolites (DHAP and GA3P) essential for de novo lipogenesis. The PPP intermediate, Xy‐5‐P, promotes ChREBP translocation to the nucleus, triggering the upregulation of genes involved in fructose metabolism and lipogenesis. Rapid phosphorylation of fructose by KHK‐C also depletes ATP, contributing to purine deamination and subsequent uric acid production. In contrast, glucose metabolism enhances glycolysis by depleting ATP levels, creating a positive feedback loop. Additionally, glucose can be converted into fructose via the polyol pathway, amplifying fructose’s effects on the liver. Enzyme activation is indicated by green pathways. ChREBP, carbohydrate response element‐binding protein; DAG, Diacylglycerol; DHAP, dihydroxyacetone phosphate; GA, glyceraldehyde; GA3P, glyceraldehyde‐3‐phosphate; GLUT‐2, glucose transporter‐2; PPP, pentose phosphate pathway.

Despite the similarities between these pathways, several differences are important for understanding the proposed survival pathway and how its chronic activation by excessive fructose consumption may contribute to MetS (Figure 2). Unlike glucose, which can be metabolized by all body cells, most cells cannot directly metabolize fructose. Instead, the metabolism of fructose occurs in the cytosol of specific cells, including those in the liver, muscle, adipose tissue, gut, and sperm [52, 53]. Fructolysis is regulated in part by FK and is also influenced by the transcription factor carbohydrate response element‐binding protein (ChREBP). The transcriptional activity of ChREBP is regulated through several mechanisms, including phosphorylation and dephosphorylation, O‐GlcNAcylation, acetylation, and the allosteric effects of various metabolites [54–56]. Fructose is metabolized into acetyl‐CoA more rapidly than glucose because fructolysis bypasses the key rate‐limiting steps of glycolysis. While the direct effect of fructose on ChREBP is weaker than that of glucose, under typical dietary conditions, fructose and glucose are often consumed together. The presence of glucose enhances the intestinal absorption of fructose compared with the absorption of fructose alone [57]. Consequently, glucose‐derived metabolites primarily activate ChREBP, which subsequently promotes fructolysis and de novo lipogenesis [51] (Figure 2).

Fructose metabolism involves rapid ATP consumption, which increases AMP degradation and uric acid production. This rise in uric acid occurs both within cells and in the bloodstream. Furthermore, uric acid may regulate fructose metabolism by affecting FK activity and activating the transcription factor ChREBP [58]. Unlike fructolysis, PFK‐1 activity in glycolysis is tightly regulated [59]. It is activated in energy‐depleted states (e.g., by AMP and fructose‐2,6‐bisphosphate) and inhibited in energy‐surplus states (e.g., by ATP, citrate, and H^+^) [60]. Since fructolysis bypasses the stringent control of PFK‐1, it remains relatively unregulated (Figure 2).

Fructose enters hepatocytes independently of insulin [61] and appears to specifically activate hepatic pyruvate dehydrogenase (PDH), at least in part by inhibiting PDH kinase [62]. This effect has been associated with decreased TCA cycle activity and ATP synthesis [63]. These findings indicate enhanced metabolite flow through hepatic PDH, resulting in acetyl‐CoA synthesis, with acetyl‐CoA subsequently being directed toward the production of fatty acids and TGs [62]. Sustained TG production may contribute to NAFLD, which may progress to insulin resistance and ultimately contribute to the development of diabetes [64].

Experimental studies suggest that inhibiting FK, the initial enzyme involved in fructose metabolism, can prevent multiple features of the MetS. This inhibition prevents the accumulation of fructose‐1‐phosphate and disrupts the turnover of adenine nucleotides [65]. Notably, liver‐specific FK knockout mice consumed significant quantities of fructose but did not develop weight gain or features of MetS [66].

Excessive glucose availability can promote its conversion into fructose through the polyol pathway (Figure 1). This process is primarily driven by the activation of a key enzyme called AR. The activity of this enzyme can also be increased by high osmolality, which may result from the consumption of salty foods [67], as well as by high‐glycemic foods [68] or alcohol [69]. Fructose metabolism may subsequently activate the proposed survival switch [70]. As a result, a high‐carbohydrate diet may contribute to the development of features associated with MetS, partly through the conversion of glucose into fructose.

At the cellular level, high fructose intake leads to cellular ATP depletion as a result of fructose phosphorylation by FK. Because the activity of aldolase B may become relatively rate‐limiting, fructose‐1‐phosphate can accumulate, further contributing to ATP depletion. As one of the three aldolase isoenzymes (A, B, and C), aldolase B plays a key role in both glycolysis and fructolysis, exhibiting similar catalytic activity toward fructose‐1‐phosphate and fructose‐1,6‐bisphosphate [71]. However, it may function as a relative rate‐limiting step in hepatic fructose metabolism, as indicated by the marked increase in hepatic fructose‐1‐phosphate levels following excessive fructose consumption [72, 73].

To better understand the importance of these pathways, one should note that an elevated fructose consumption initially results in hepatic ATP depletion, ending up with an increased ADP and AMP, accumulation of fructose 1‐phosphate, and a decrease in inorganic phosphate due to its sequestration within fructose 1‐phosphate. However, it should be considered that the reduction in ATP cannot be fully attributed to a proportional rise in ADP and AMP. Instead, ATP depletion is largely associated with an increased uric acid production [74].

When ATP levels decline, cellular mechanisms act to preserve the energy balance by reducing ADP levels. High fructose consumption can cause an accumulation of fructose 1‐phosphate, which disrupts the ATP/ADP ratio, a crucial factor in the regulation of survival pathways, leading to the conversion of ADP to AMP [75, 76]. AMP is then converted to ammonia by AMP deaminase‐2 (AMPD2). Ultimately, this pathway increases uric acid production [75, 76].

**Figure 3:**
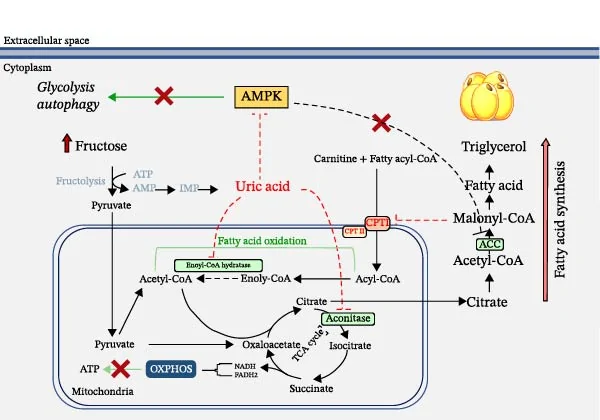
Fructose stimulates hepatic uric acid synthesis. Fructose stimulates the synthesis of hepatic uric acid by being quickly phosphorylated in the liver, resulting in ATP depletion and AMP accumulation. This process activates AMP deaminase, which converts AMP into IMP and ultimately uric acid. Uric acid has been shown to suppress the activity of AMPK, enoyl‐CoA, and aconitase, which impacts various metabolic pathways. AMPK acts as a negative regulator of ACC activity. Active ACC catalyzes the conversion of acetyl‐CoA into malonyl‐CoA. Malonyl‐CoA allosterically inhibits CPT‐1, which facilitates the transfer of the fatty acyl group from acyl‐CoA to carnitine, preparing it for transport from the cytosol into the mitochondria, where it is utilized for β‐oxidation. ATP, adenosine triphosphate; AMP, adenosine monophosphate; AMPK, AMP‐activated protein kinase; ACC, acetyl‐CoA carboxylase; CPT‐1, carnitine palmitoyltransferase‐1; IMP, inosine monophosphate.

Uric acid induces mitochondrial oxidative stress by promoting the translocation of NADPH oxidase to the mitochondria [77]. It disrupts fatty acid oxidation by inhibiting enoyl‐CoA hydratase and aconitase activity in the TCA cycle [78]. Furthermore, uric acid also inhibits AMP‐activated protein kinase [76]. As a result, the cell shifts into an energy‐conserving state, decreasing both ATP production and consumption, which keeps intracellular ATP levels low [79] (Figure 3). The decrease in intracellular ATP levels may serve as a signal that activates pathways associated with multiple features of the MetS [80–82].

In summary, although glucose and fructose share overlapping metabolic pathways, their metabolic consequences differ substantially. Fructose metabolism bypasses key regulatory steps of glycolysis, leading to rapid acetyl‐CoA and lipid synthesis. Excessive fructose intake depletes intracellular ATP, promotes uric acid production, and disrupts the cellular energy balance. Uric acid exacerbates oxidative stress, inhibits mitochondrial function, and impairs metabolic regulation. These combined effects may activate the proposed survival pathway and contribute to the development of MetS.

## 5. Fructose, Uric Acid, and Vasopressin: Key Players in MetS

Individuals with MetS or related conditions, such as NAFLD, frequently exhibit elevated levels of vasopressin, as reflected by increased levels of its surrogate marker, copeptin [83, 84]. Research has demonstrated that fructose significantly influences vasopressin secretion [85]. Specifically, intravenous administration of fructose stimulates vasopressin release, unlike the administration of iso‐osmolar glucose, and enhances vasopressin synthesis in hypothalamic explants via the action of FK [86].

Water and salt balance is closely linked to vasopressin regulation as elevated serum osmolality stimulates vasopressin secretion. This increase in vasopressin subsequently activates AR in the liver, resulting in the production of endogenous fructose [67]. Additionally, both acute and chronic dehydration can stimulate AR activity in the hypothalamus, resulting in local fructose production, which contributes to the vasopressin response [86]. A high‐salt diet may worsen this effect by increasing fructose and uric acid production [67]. This response may be particularly important because both high‐salt and high‐fructose intake increase vasopressin levels. The vasopressin V2 receptor is essential for urine concentration through its role in promoting water reabsorption in the renal collecting ducts [87]. In contrast, the vasopressin V1a receptor contributes to vasoconstriction and the regulation of blood pressure, thereby playing a key role in the development of hypertension [88]. Additionally, the vasopressin V1b receptor has been implicated in fructose‐induced MetS by modulating hypothalamic–pituitary–adrenal (HPA) axis activity and influencing glucose and lipid metabolism [82, 88]. In summary, these findings indicate interactions among fructose, uric acid, and vasopressin in the hormonal regulation of the MetS.

The survival switch refers to a biological response that promotes energy storage and metabolic adaptations in anticipation of environmental stress or resource scarcity [89]. The activation of this mechanism prepares the organism for periods of limited food or water availability. Its activation is associated with increased body weight, enhanced fat and glycogen stores, insulin and leptin resistance, elevated blood pressure, salt sensitivity, and low‐grade systemic inflammation [39]. These physiological changes may support survival by augmenting energy reserves necessary for hibernation, long‐distance migration, nesting, or other scenarios in which access to food, water, or oxygen is restricted [90–95].

Additionally, certain protein‐rich foods, especially those found in red and processed meats, may increase the risk of MetS [96–99]. One proposed explanation is that these meats and shellfish are high in glutamate and nucleotides such as inosine monophosphate (IMP) and adenosine monophosphate (AMP) [100]. IMP plays an important role in the fructose‐associated energy‐depletion pathway, while glutamate can be converted into uric acid in the liver. Studies suggest that umami compounds, including glutamate and IMP, may increase uric acid production, resulting in hepatic ATP depletion and contributing to MetS through mechanisms resembling those associated with fructose [100, 101].

In modern society, resource scarcity is less common, and metabolic regulation may instead be disrupted by (1) excessive fructose and sugar intake; (2) high‐salt diets that activate the polyol pathway, increasing endogenous fructose production; and (3) umami foods rich in glutamate and purines (such as AMP and IMP), which stimulate FK [102]. Thus, the three tastes, sweet [103], salty [67], and umami [100], evolved to encourage food intake for energy storage and survival. However, excessive consumption of these tastes can lead to MetS through similar mechanisms [67, 100, 103].

Humans have experienced a dramatic increase in the consumption of added sugars, particularly those containing fructose and glucose, such as table sugar (sucrose) and HFCS. Humans also have higher uric acid levels than most other mammals because of a mutation in the uricase gene that occurred during the mid‐Miocene [104]. This loss of uricase in early hominoids resulted in an amplified uric acid response to fructose, thereby enhancing the stimulation of fat and glucose production by a given fructose dose [102]. The uricase mutation has been proposed to have conferred a survival advantage during periods of global cooling and food scarcity [105], potentially supporting survival under these conditions. Consequently, the loss of uricase may have functioned as a “thrifty gene,” as proposed by James Neel in 1962 [106], enhancing survival during food shortages but potentially contributing to MetS in modern societies.

Notably, this proposed survival mechanism may be intermittently activated in humans depending on the quantity and speed of ingestion, as well as genetic and environmental factors. Notably, whole fruits typically do not trigger this pathway due to their relatively low fructose density and the presence of protective components, including fiber, vitamin C, potassium, and flavanols [70]. The fiber in fruit slows the release of fructose, allowing the small intestine to gradually convert it into glucose. This process helps reduce the fructose load in the portal vein before it reaches the liver and other tissues [70, 107].

## 6. The Energy Reduction–Oxidation Model of Obesity

At the mitochondrial level, when cellular energy requirements are satisfied but excess fuel remains available, the mitochondria generate reactive oxygen species (ROS) as signaling molecules that promote fuel storage by stimulating insulin secretion [108]. Under physiological energy conditions, once substrates are stored and no surplus glucose or lipids remain, mitochondria utilize reduced nicotinamide adenine dinucleotide (NADH) to drive the electron transport chain (ETC), thereby maintaining a high‐energy state, as indicated by an elevated ATP/ADP ratio [109].

**Figure 4:**
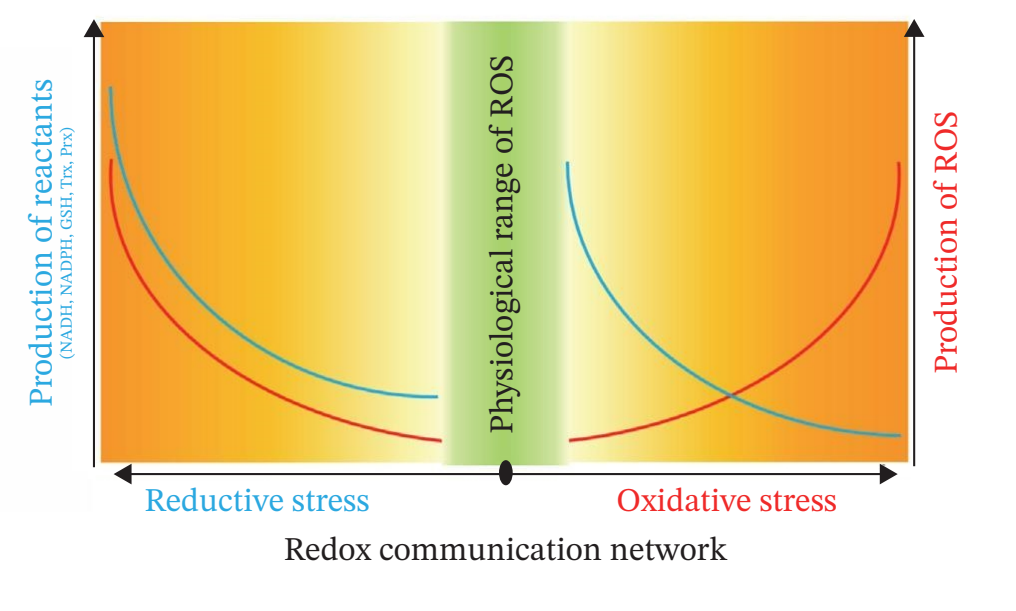
The redox communication networks. Redox components are essential for mediating electron transfer between reduced and oxidized compounds. Key participants indicating redox state include NAD^+^, NADP^+^, oxidized glutathione (GSSG), thioredoxin (Trx^o^x), and peroxiredoxin (Prx^o^x) in their oxidized forms, along with NADH, NADPH, glutathione (GSH). thioredoxin (Trx), and peroxiredoxin (Prx) in their reduced forms.

However, in the presence of energy excess, the intracellular levels of NADH and ROS increase markedly across tissues [110]. Conversely, during energy deficiency, both NADH and ROS concentrations decline. Elevated ROS levels are neutralized by NADPH, which is produced through glucose and fat metabolism. NADPH is regenerated from NADH by the enzyme nicotinamide nucleotide transhydrogenase (NNT), which plays a crucial role in ROS neutralization via its interaction with peroxidase systems [111]. Collectively, the redox network, comprising NADH/NAD^+^, NADPH/NADP^+^, and GSH/GSSG, functions as an energy‐sensing communication system across cellular and subcellular compartments [109]. A shift toward increased reducing equivalents leads to a state known as reductive stress. In contrast, oxidative stress is characterized by an imbalance favoring oxidant production, including excessive ROS accumulation. Reductive stress has been described as a state of “over‐electron leakage,” analogous to water spilling over from a dam when capacity is exceeded [112] (Figure 4).

The redox couples are compartmentalized into distinct mitochondrial and cytosolic environments, each characterized by unique redox potentials [113]. Within mitochondria, elevated levels of NADH support ATP generation via oxidative phosphorylation, maintain a negative mitochondrial membrane potential, and facilitate the production of NADPH for ROS detoxification [114]. In contrast, low cytosolic NADH concentrations promote glycolysis [109, 115, 116], while elevated cytosolic NADPH levels support anabolic processes, such as de novo lipid synthesis, and sustain the cellular antioxidant defense machinery [109]. Additionally, thiol‐disulfide couples are maintained in a reduced state in both the mitochondrial and cytosolic compartments to safeguard against oxidative stress [109]. Taken together, disruptions in this redox compartmentalization and signaling can impair metabolic flexibility and contribute to the development of cellular metabolic disorders, including insulin resistance and steatosis.

## 7. Electron Versus Proton Leakage: NNT’s Role in Mitochondrial Energy Dynamics and ROS Management

During oxidative phosphorylation, electrons derived from metabolic fuels such as glucose and fatty acids are transferred through the mitochondrial ETC located in the inner mitochondrial membrane, ultimately reducing molecular oxygen to water [117]. According to Peter Mitchell’s [118] chemiosmotic theory (1961), the energy released from electron transfer establishes a proton gradient across the inner membrane, which drives ATP synthesis by coupling respiratory oxygen consumption with ADP phosphorylation.

**Figure 5:**
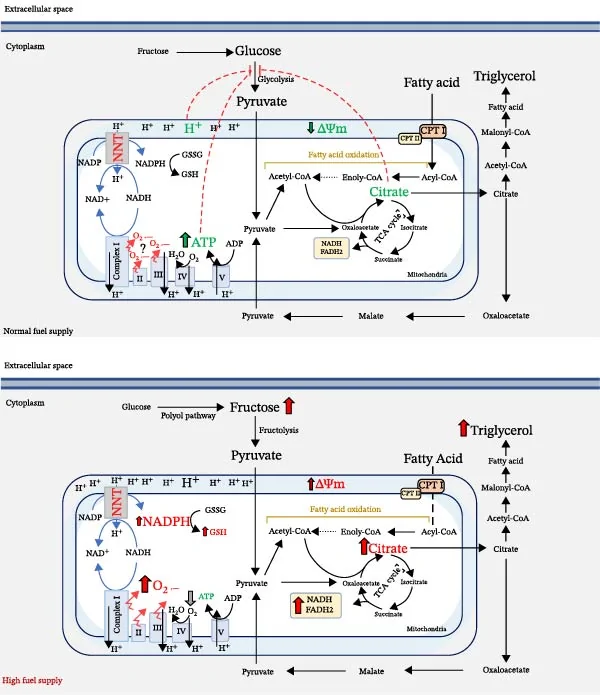
How mitochondria produce O_2_•−, influenced by NADH/NAD^+^ ratios and ATP demand. Normal fuel supply: ATP is actively produced, leading to a lower proton gradient and a more oxidized NADH pool, which reduces O_2_•− production. High fuel supply: Mitochondria shift into two modes that significantly increase O_2_•− production, primarily from Complex I: (i) when ATP production is absent, resulting in a high proton motive force (Δ*p*) and a reduced CoQ pool, and (ii) when the NADH/NAD^+^ ratio in the mitochondrial matrix is elevated. O_2_•− ‐ Superoxide, ATP ‐ Adenosine Triphosphate, p ‐ Proton motive force, CoQ ‐ Coenzyme Q, NADH ‐ Nicotinamide Adenine Dinucleotide (reduced form), NAD^+^ ‐ Nicotinamide Adenine Dinucleotide (oxidized form), Δ*p* ‐ Proton gradient, NNT: Nicotinamide nucleotide transhydrogenase.

Mitochondrial ROS production is closely linked to NNT‐mediated ROS removal. NNT plays a critical role in mitochondrial homeostasis by producing mitochondrial NADPH, a key molecule for ROS scavenging. By maintaining the thiol redox state, NNT provides cellular protection; however, its function relies on the proton gradient, resulting in a degree of proton leak during NADPH synthesis. In essence, NNT utilizes excess reducing equivalents to generate NADPH, thereby limiting ROS accumulation at the expense of some energy loss [110, 119, 120] (Figure 5).

Under physiological energy conditions, proton leakage associated with mitochondrial NNT activity (i.e., conversion of NADH to NADPH) occurs concurrently with electron leakage to oxygen, producing basal levels of ROS. This controlled ROS production is crucial for sustaining a high‐energy state, reflected in an elevated ATP/ADP ratio, which is essential for optimizing energy efficiency, stabilizing fuel reserves, and managing body weight [110]. In contrast, in states of energy surplus, the ROS neutralization increases NNT flux, leading to increased energy dissipation, effectively “wasting” energy [110]. This process, although energetically inefficient, supports energy homeostasis by preventing excessive fuel storage [110]. However, overloading this ROS management system in response to persistent caloric excess may impair the mitochondrial energy efficiency, maintain heightened ROS levels, and contribute to oxidative damage and metabolic dysregulation [121] (Figure 5). These findings underscore the importance of NNT as a central metabolic regulator that balances energy expenditure and redox stability. Dysregulation of this system may predispose cells to metabolic disorders, including insulin resistance, steatosis, and obesity‐related complications.

## 8. Chronic Activation of Survival Pathways and Persistent ROS Elevation

Excessive levels of ROS can result not only from acute oxidative stress but also from prolonged activation of survival pathways or over‐supplementation with antioxidant agents/molecules. Unlike acute/transient ROS elevation, chronic ROS accumulation persists even in metabolically active cells, despite ongoing cellular damage such as lipid peroxidation, protein denaturation, and endoplasmic reticulum (ER) stress [122]. These chronically elevated ROS specifically stimulate the following processes:

1. Adipocyte fat storage: The storage of TGs in adipocytes is tightly regulated by ROS and modulated by the hormonal milieu. Insulin promotes TG synthesis and storage by stimulating vascular lipoprotein lipase (LPL) and inhibiting lipolysis [123, 124]. Conversely, catecholamines trigger lipolysis, releasing free fatty acids and glycerol into the circulation [125, 126]. Chronic hyperinsulinemia increases TG accumulation, while low basal insulin levels are associated with reduced TG deposition.
2. Pancreatic β‐cells: In pancreatic β‐cells, both exogenous application and endogenous generation of ROS stimulate insulin secretion [108, 127, 128].
3. Hepatic glucose regulation: Hepatic glucose output is reduced by a physiological increase in ROS, regardless of whether the ROS originate from the mitochondria, the cytosol, or extracellular sources [129].
4. Hypothalamic regulation of food intake: Within the hypothalamus, ROS modulate neuronal activity to regulate appetite [130]. Pathologically elevated ROS levels favor the production of β‐endorphin, an orexigenic peptide, over α‐melanocyte‐stimulating hormone (α‐MSH), an anorexigenic peptide derived from the pro‐opiomelanocortin (POMC) precursor [131, 132]. This neuropeptide imbalance promotes hyperphagia and contributes to increased energy intake [132].

While antioxidant supplementation may enhance the cellular antioxidant capacity, excessive supplementation may induce reductive stress, a condition characterized by excessive reducing equivalents [112]. In obesity, chronic overnutrition and the consumption of ultra‐processed foods (UPFs) may reduce cellular antioxidant capacity [133], potentially leading to increased ROS generation and persistent activation of survival pathways (Figure 4).

In nature, the seasonal cycle of summer, followed by fall, demonstrates the evolutionary advantage of the “survival pathway,” which enhances energy storage in preparation for scarcity. However, in modern environments with continuous access to abundant calorie‐dense food, this pathway may become chronically activated. This persistent activation, likely initiated by reductive stress due to sustained nutrient excess, is implicated in the pathogenesis of MetS via ROS‐mediated cellular dysfunction. Notably, reductive stress may represent a transient state that can evolve into oxidative stress, especially as cellular damage and mitochondrial dysfunction accumulate [134]. Within tissues, certain cell types may remain in a reductive state, while others progress to oxidative stress, leading to heterogeneous metabolic consequences [134].

Nutrient availability in the circulation is primarily determined by dietary intake, with metabolite concentrations varying according to absorption and meal composition. Fructose, for instance, can increase in plasma 7‐ to 10‐fold following ingestion [135]. Its intestinal absorption becomes more efficient with repeated exposure as transport across the intestinal epithelium is upregulated [136]. Western diets, characterized by a high intake of sugar and fat, have been strongly associated with metabolic diseases [137] and male infertility [44]. Given its abundance in such diets, fructose is increasingly implicated in the etiology of both obesity and reproductive dysfunction [44, 137]. In addition to dietary intake, fructose is endogenously synthesized via the polyol pathway, in which glucose is first reduced to sorbitol and subsequently oxidized to fructose. This pathway is particularly active in male reproductive tissues [138]. However, its role in male fertility remains poorly understood. A prominent hypothesis suggests that under diabetic or hyperglycemic conditions, the polyol pathway becomes upregulated in the testes, potentially contributing to impaired reproductive function and infertility [44].

In summary, while ROS serve essential signaling roles under physiological conditions, chronic elevation resulting from sustained activation of survival pathways, often triggered by overnutrition and excess nutrient availability, may lead to substantial metabolic disturbances. This persistent oxidative burden disrupts energy homeostasis, promotes adiposity, alters insulin secretion, impairs hepatic glucose regulation, and dysregulates hypothalamic appetite control. Moreover, the interplay between reductive and oxidative stress, particularly in the context of modern Western dietary patterns rich in fructose and UPFs, contributes to the development of MetS and may adversely affect male reproductive health. Understanding these mechanisms highlights the importance of metabolic flexibility and redox balance in maintaining cellular and systemic health.

## 9. The Polyol Pathway in the Male Reproductive System

The testes are paired male gonads organized into distinct compartments, with seminiferous tubules surrounded by the connective tissue and interstitial Leydig cells. The epithelium of the seminiferous tubules is organized by Sertoli cells (SCs), first identified by Enrico Sertoli in 1865. SCs are critical for sex determination during fetal development and support germ cell maturation in adulthood. Their role begins during the early developmental stages as the primordial gonads differentiate into the male phenotype through SC differentiation, which regulates the migration, proliferation, differentiation, and function of other testicular cell types. Thus, the SCs are indispensable for the proper functioning of the testis throughout fetal development and postnatal life [139].

Within the testis, the expression of AR and SDH varies by cell type [138]. AR is predominantly expressed in SCs and, to a lesser extent, in spermatogenic cells [138]. However, spermatozoa in the seminiferous tubules do not express AR. In contrast, SDH is present in all developing germ cells and reaches its highest levels during spermatid elongation, after which it is transferred to spermatozoa [138].

Research has shown that spermatozoa in the epididymis contain substantial levels of both AR and SDH proteins. Consistent with this finding, the epididymal epithelium produces epididymosomes, which transfer proteins to spermatozoa, including key enzymes from the polyol pathway [140]. Consequently, the cauda epididymis shows a higher AR expression than the caput, while the SDH levels remain relatively constant. The presence of polyol enzymes in the male reproductive tract serves functions beyond fructose production; for instance, AR localization in the SCs and epididymis is believed to be functionally significant [140]. Additionally, these enzymes may protect the spermatozoa against numerous toxic substances encountered during their transit through the male and female reproductive tracts [141]. Evidence from studies in other tissues supports the hypothesis that AR acts as a “cellular guardian” against oxidative stress [142], a role similarly observed in vascular smooth muscle cells [143] and cardiac myocytes [144].

AR likely contributes to the detoxification of reactive carbonyl compounds, including methylglyoxal, a by‐product of glucose metabolism, and 4‐hydroxynonenal, a lipid‐derived aldehyde formed during oxidative stress [142]. Under conditions of increased glucose influx, the excess glucose is shunted into the polyol pathway, where AR converts it to sorbitol using NADPH as a cofactor [145, 146]. This reaction depletes NADPH, a crucial antioxidant coenzyme, leading to impaired regeneration of reduced glutathione (GSH) and heightened oxidative stress [147].

The aldo‐keto reductase (AKR) superfamily plays a pivotal role in neutralizing harmful carbonyl compounds, indicating that AR in SCs may contribute to the detoxification of toxic metabolites generated during spermatogenesis [138]. Moreover, since sorbitol is found in both uterine fluid and seminal plasma, the SDH in spermatozoa likely converts sorbitol into fructose, providing a crucial energy source for spermatozoa [148].

The composition of uterine fluid, particularly its fructose concentration, plays a direct role in sperm capacitation [149]. AR has been identified in the endometrium, supporting the hypothesis that its expression in the endometrial epithelium regulates the uterine environment and facilitates sperm capacitation [150]. Moreover, glucose and fructose concentrations in uterine fluid, along with AR expression, have been shown to vary cyclically in association with fluctuations in uterine glucose and fructose levels during the estrous cycle and pregnancy [150]. Notably, inhibition of AR in the endometrium reduces uterine fructose levels, thereby impairing sperm capacitation and fertilization [150].

Taken together, the polyol pathway, mediated by AR and SDH, plays essential roles in male reproductive physiology, extending beyond fructose production to include sperm maturation, energy metabolism, and protection from oxidative stress. These enzymes are dynamically regulated within the testes and epididymis and are also functionally active during sperm transit through the female reproductive tract. Moreover, their involvement in regulating the uterine environment underscores the complex interplay between male and female reproductive systems. Dysregulation of the polyol pathway, particularly through altered glucose metabolism and NADPH depletion, may compromise the redox balance, impair spermatogenesis, and ultimately contribute to male infertility. These observations provide a basis for examining the specific role of fructose in seminal plasma.

## 10. Seminal Plasma Fructose

Fructose is an important component of human semen. Pioneering research by Ivanov [151], and Huggins and Johnson [152], established that semen contains a reducing substance, laying the foundation for the field of semen biochemistry. While this substance was initially misidentified as glucose, Mann [153] successfully demonstrated in 1946, through chemical analyses, that the sugar present in the semen was actually fructose. Mann conducted his research primarily on animal semen, but his findings had important implications for the biochemistry of human semen. Mann’s identification of fructose in semen opened a new area of investigation in reproductive biochemistry and human fertility research.

Fructose serves as both an anaerobic and aerobic energy source for sperm and has been indirectly linked to sperm vitality and progressive motility [154]. As a result, a reduced sperm count, decreased motility, and a higher proportion of morphologically abnormal sperm may lead to fructose accumulation in semen. Consequently, a negative correlation has been observed between the concentration of fructose in seminal plasma and the motile sperm count [155]. Seminal fructose levels were higher in men with obstructive azoospermia and in those who remained azoospermic after vasoepididymostomy performed to treat epididymal blockage [156]. Fructose is recognized as a vital bioenergetic substrate for spermatozoa, and its availability may reflect reproductive adaptations that support sperm survival. During the breeding season, seminal plasma fructose concentrations increase in seasonal breeders, highlighting its physiological relevance during periods of increased fertility [157]. It is believed that fructose is synthesized from blood glucose by the accessory sex glands, which are stimulated by testosterone [157].

In contrast to fructose, glucose is the primary energy source for spermatozoa within the female genital tract, reaching its highest concentration in cervical mucus during ovulation [158]. Given that fructose is important for spermatozoa bioenergetics and is likely synthesized from glucose under testosterone stimulation, with levels peaking during the breeding season [157], it is plausible to suggest that these mechanisms are optimized to support fertility during specific reproductive windows. Given that obesity is associated with metabolic dysfunction and may involve chronic activation of survival pathways, Kozopas et al. [159] found that the seminal plasma of pre‐obese and obese men exhibited reduced fructose levels compared with men of normal weight, despite poorer sperm parameters. However, the data on this topic remain controversial. For instance, Luque et al. [160] reported reduced seminal fructose levels among morbidly obese men. In contrast, other studies have found either a positive correlation [161] or no significant relationship between body mass index (BMI) and seminal fructose concentrations [162, 163].

## 11. Influence of Fructose on Testicular Development

Studies have indicated that adequate glucose availability is important for testicular development [164, 165]. Disruption of glucose metabolism induced by excessive fructose consumption may adversely affect early‐stage testicular development. Supporting this possibility, Shibata and Fukuwatari [166] reported the effects of high‐fructose diets containing 70% or 80% fructose on body weight, organ development, and metabolism in weaning rats under both low‐protein (10% casein) and normal‐protein (20% casein) conditions. They found that rats fed an 80% fructose diet at the 10% protein level exhibited a markedly reduced body weight gain (36% of that observed in the control group). Food intake was also significantly lower, at 46% of the control group’s intake. Among organ weights, the testes were the most sensitive, showing a 37% reduction under low‐protein conditions and a 19% reduction under normal‐protein conditions. The study suggested that fructose does not stimulate insulin secretion, which may contribute to decreased food intake and disrupted metabolic regulation. In addition, plasma markers in rats fed diets containing ≥56% fructose showed significantly increased TGs, non‐esterified fatty acids (NEFA), and total ketone bodies, indicating enhanced lipid metabolism. They concluded that insulin may play a pivotal role in the cascade of events leading to increased food intake. In the absence of glucose, the insulin levels remain very low. Given that leptin regulation is dependent on insulin, the sequence of satiety‐inducing signals may be disrupted [167]. Therefore, these findings suggest that highly enriched fructose diets may adversely affect male reproductive development. Consistent with the reduction in testicular weight, the plasma testosterone levels decreased significantly by 47.9% following 4 weeks of fructose feeding compared with those in control rats [168]. Investigating how dietary fructose contributes to testosterone deficiency may therefore represent an important direction for future research. The observed reduction in circulating testosterone levels may be attributed to impaired testicular development, as noted after 4 weeks of fructose consumption in this study. Previous research has highlighted the importance of adequate glucose levels for normal testicular development [164]. Consequently, an altered glucose metabolism resulting from fructose intake may have adversely affected testis growth during the early developmental stages.

Therefore, this raises an important question: could the observed reduction in sperm count be linked to aberrant testicular development associated with a high‐fructose diet? This hypothesis warrants further investigation and opens several avenues for exploration. For instance, could low protein intake due to socioeconomic status influence fertility outcomes? Additionally, might adherence to a vegetarian or vegan diet impact fertility status?

Considering previous reports and these questions, impaired testicular development associated with altered glucose metabolism and insufficient insulin signaling may contribute to the reproductive effects of high‐fructose intake. Furthermore, low protein intake, potentially linked to socioeconomic factors, may exacerbate these effects by limiting the availability of essential amino acids required for normal reproductive organ growth. In addition, plant‐based diets such as vegetarian or vegan regimens may influence fertility outcomes depending on protein quality and adequacy, particularly in the context of high‐dietary fructose consumption [169]. These possibilities should be considered hypotheses rather than established causal relationships and require direct investigation in appropriately controlled studies. Collectively, they emphasize the need to examine how fructose exposure interacts with the overall dietary quality during testicular development.

## 12. Insulin Resistance and Testicular Development

Insulin is not only vital for systemic metabolic regulation but also plays a key role in reproductive physiology. In particular, insulin signaling is essential for the proper development and function of the testes [170]. This signaling is mediated through a family of insulin receptor (IR) tyrosine kinases that are involved in various cellular and developmental processes [171]. Disruptions in these pathways can have profound effects on the testicular structure, hormone production, and spermatogenesis.

More specifically, the IR tyrosine kinase family comprises the IR, the insulin‐like growth factor 1 receptor (IGF‐1R), and the insulin‐related receptor (IRR). These receptors are ubiquitously expressed in mammalian cells and are crucial for numerous physiological processes [172]. Notably, they play a critical role in the proper development and function of the testis [173–175]. Within the testis, insulin signaling is mediated by pathways involving IR substrates (IRS‐1/2) and the PI3K–Akt–mTOR signaling cascade [176, 177].

IRS‐2 is an intracellular protein that undergoes phosphorylation upon IR activation. Its downregulation has been shown to impair testis development, while complete deletion of IRS‐2 induces peripheral insulin resistance [178]. Therefore, deletion of IRS‐2 results in a significant reduction in testicular weight, accompanied by reductions in the numbers of spermatocytes and SCs [178]. Loss of IRS‐2 has been associated with progressive testicular atrophy, primarily affecting germ cells within the seminiferous tubules. Accordingly, in IRS‐2−/− mice, abnormal cells are frequently observed in the testicular epithelium along with irregularities in the spermatogonia [178]. Furthermore, these mice exhibit significantly reduced cell proliferation and increased rates of apoptosis [178].

In this context, Yildirim et al. [47] demonstrated that a high‐fructose diet induces testicular insulin resistance by suppressing the downstream molecules within the PI3K‐Akt‐mTOR pathway, a central axis in insulin signaling. These observations are consistent with similar effects reported in diabetic animal models, where insulin resistance affects various organs, including the liver [179], adipose tissue [180], blood vessels [181], and kidneys [182], thereby underscoring insulin’s role as a key regulatory mediator. The study also highlighted the association between type 2 diabetes, obesity, and hypogonadotropic hypogonadism, suggesting a shared pathophysiological basis [183]. Importantly, Yildirim et al. [47] further revealed a close interdependence between mTOR and insulin signaling pathways within the testis, both of which were suppressed by a high‐fructose diet.

Beyond these findings, interactions between insulin signaling and reproductive hormones should also be considered. Given that follicle‐stimulating hormone (FSH) is a major physiological regulator of the reproductive system, it promotes SC proliferation via the cAMP/PKA/ERK and PI3K/Akt/mTORC1 pathways and governs SC differentiation and apoptosis via the cAMP/PKA pathway [184]. Consistent with this relationship, Chosich et al. [185] demonstrated that insulin resistance, when combined with elevated lipid levels, acutely suppresses FSH secretion, suggesting a potential mechanism for the relative hypogonadotropic hypogonadism often observed in individuals with obesity. Consequently, the detrimental impact of a high‐fructose diet, which promotes insulin resistance, may significantly disrupt these critical hormonal and intracellular signaling pathways.

Furthermore, studies have shown that the expression of endothelial nitric oxide synthase (eNOS), a protein activated by insulin signaling, is essential for maintaining adequate blood flow to organs [47]. In rats fed a high‐fructose diet, eNOS expression was markedly reduced in the testes, which may be linked to insulin resistance, a phenomenon that parallels observations in diabetes and MetS [186]. Within the testis, the eNOS protein has been localized to the cytoplasm of Leydig and SCs across all stages of spermatogenesis and to the epithelial layers of the epididymis and vas deferens [186]. Additionally, eNOS is expressed in endothelial cells across all tissues, though notably absent in normal germ cells. However, eNOS activity has been detected in degenerating or apoptotic intraepithelial germ cells [187]. The expression of eNOS in SCs appears to be influenced by the presence of germ cells and is likely associated with their development [188]. This broad distribution suggests that eNOS may play a vital role in both normal and abnormal testicular function.

Taken together, these findings suggest that the crosstalk between insulin, eNOS, and hormonal signals highlights how insulin resistance and metabolic dysfunction—particularly those induced by a high‐fructose diet—profoundly impair male fertility through multiple, interconnected insulin‐related pathways.

## 13. Influence of Fructose on the Blood–Testis Barrier (BTB)

The BTB is a highly specialized structure formed by the interactions of tight junctions (TJs), adherens junctions, desmosomes (DSs), and gap junctions between SCs, playing a pivotal role in maintaining the unique environment necessary for spermatogenesis [189]. The TJs, in particular, are composed of key transmembrane and cytoplasmic proteins, including occludin, claudins, zonula occludens (ZO‐1, ZO‐2, and ZO‐3), and junctional adhesion molecules, which collectively ensure the integrity of epithelial barriers across various tissues [190, 191].

Among these, ZO proteins act as essential cytoplasmic adaptor molecules linking transmembrane TJ proteins to the actin cytoskeleton in epithelial and endothelial cells, thereby contributing to structural stability at cell junctions [192]. Occludin and claudins, as principal transmembrane proteins, are instrumental in the formation of barriers in epithelial and endothelial tissues [193]. Claudins, in particular, are directly responsible for establishing paracellular ion‐selective channels or pores, determining the selective permeability and physiological specificity of the barrier [194].

Numerous studies indicate that dietary fructose exerts a deleterious effect on epithelial barrier function, including BTB integrity [195, 196]. In this regard, Akar et al. (2022) demonstrated that excessive fructose intake downregulates the expression of claudin‐11 and N‐cadherin, as well as SIRT1, in the testicular tissue of rats. These changes are associated with testicular degeneration and disorganization [195]. Additionally, high‐fructose diets are known to trigger inflammatory responses, marked by cytokine activation and histological evidence of vacuolar and intratubular degeneration, necrotic debris accumulation, and disruption of the seminiferous tubules [197]. These pathological changes not only compromise the physical integrity of the BTB but may also lead to hormonal dysregulation, including decreased intratesticular testosterone levels. This hormonal imbalance, in turn, has significant implications for male fertility, highlighting the critical intersection between metabolic health, barrier function, and reproductive capacity [195, 197].

## 14. Influence of Fructose on Leydig Cells

Apoptotic Leydig cells and elevated inflammatory markers observed in the testes of fructose‐fed rats provide further evidence supporting the established link between decreased plasma testosterone levels and key MetS components, such as hyperglycemia, insulin resistance, and dyslipidemia [198–203]. These pathological changes highlight the broader systemic effects of metabolic dysregulation on the male reproductive function. Within the testicular environment, macrophages constitute a heterogeneous immune cell population characterized by distinct niche‐specific phenotypes and functional roles [204]. Beyond their classical immune functions, testicular macrophages contribute to several immune and non‐immune roles [205], including the regulation of germ cell development in coordination with SC activity [206].

In this regard, Yildirim et al. [47] demonstrated that fructose activates macrophage‐related factors (CD163 and CD68) and apoptotic markers (caspase‐8 and caspase‐3) in rat testes. This finding is consistent with a study reporting an increased number of CD68‐ and CD163‐positive macrophages in the testes of individuals with idiopathic non‐obstructive azoospermia [207]. These results suggest that the activation of CD163‐positive macrophages and apoptotic pathways may inhibit the insulin‐dependent PI3K/AKT signaling pathway, which is susceptible to disruption by excessive fructose intake [47].

These findings highlight that high‐fructose intake induces testicular macrophage activation and apoptosis, thereby disrupting insulin‐dependent PI3K/AKT signaling. This disruption may impair the function of Leydig and germ cells and reduce testosterone production. Consequently, the interaction between metabolic dysregulation and immune imbalance may compromise male fertility.

## 15. Taste Perception: Journey From the Tongue to the Testes

In mammals, the sense of taste plays a crucial role in assessing nutrient content, guiding food intake, and avoiding harmful substances. The five basic tastes—salty, sour, bitter, sweet, and umami—are detected by specialized cell types expressing distinct receptors. Of particular interest, the detection of sweet, bitter, and umami tastes involves two families of G‐protein‐coupled receptors, T2Rs and T1Rs [208]. Interestingly, these taste receptors are not confined to the tongue but are also expressed in various extraoral tissues, including the digestive and respiratory systems, brain, testes, and spermatozoa [208, 209]. However, their precise physiological roles in these non‐gustatory sites remain to be fully elucidated [209].

The taste receptor type 1 member 3 (TAS1R3) is a G protein‐coupled receptor that forms part of the heterodimeric receptor complexes responsible for detecting sweet and umami tastes [210, 211]. Emerging evidence suggests that TAS1R3 can be activated by components of the Western diet, which is typically high in saturated fat and sugary beverages [212]. To further investigate the role of TAS1R3 in the male reproductive system, Seong et al. [213] examined the function of this receptor in testicular physiology using a mouse model lacking the TAS1R3 gene. Their study revealed that testicular TAS1R3 responds to nutrient cues associated with the Western diet. Long‐term consumption of the Western diet impaired sperm quality and testicular architecture; however, these adverse effects were not observed in TAS1R3‐knockout mice. Transcriptomic analyses further revealed a metabolic shift in the knockout mice, characterized by upregulation of phosphatidylcholine biosynthesis and downregulation of AMPK signaling, alongside elevated expression of the transcription factor NR4A1. Notably, NR4A1 regulates the expression of steroidogenic enzymes and is modulated by the AMPK pathway [213].

Consistent with these findings, TAS1R3 expression was found to be enriched in Leydig cells and late‐stage spermatids [214]. To further explore this mechanism, Seong et al. [213] used siRNA to knock down TAS1R3 expression in a Leydig cell line. They demonstrated that exposure to fructose, glucose, and palmitate—nutrient components commonly found in the Western diet—compromised testosterone biosynthesis via dysregulation of the AMPK/NR4A1 pathway and suppression of key steroidogenic genes, including Star, Cyp11a1, Cyp17a1, Hsd3b1, and Hsd3b6. They concluded that the Western diet can alter gene expression in the testes and hormonal balance through TAS1R3‐mediated signaling pathways [212]. A notable limitation of this study was the lack of distinction between the individual effects of fructose, glucose, and palmitate, which may have differential impacts on testicular function. Nevertheless, analysis of the GEO database supported the clinical relevance of TAS1R3 in reproductive function, underscoring its potential as a molecular link between diet and male fertility [213].

The study by Seong et al. [213] reinforces and extends previous findings that MetS is associated with lower levels of FSH, luteinizing hormone (LH), and testosterone, thereby compromising reproductive health in both humans and animal models. These hormonal declines underscore the detrimental impact of MetS on male fertility [45], potentially due to increased total oxidative stress (TOS) and decreased total antioxidant status (TAS) in seminal fluid [215]. In the context of MetS, there is evidence of upregulated mRNA expression of several genes involved in mitochondrial function, including MFN2, PPARG, PGC1α, TXNL4B, and PARP2 in the testicular tissue of rats. These data suggest that MetS, potentially induced by high‐fructose consumption, disrupts mitochondrial dynamics and may impair testicular functions [216].

In addition, mitochondrial dysfunction associated with MetS has been linked to increased mitochondrial DNA (mtDNA) damage, particularly in the liver of rats fed a high‐fructose diet. This damage, as indicated by alterations in the expression of DNA polymerase γ, remained unrepaired and resulted in a significant reduction in the mtDNA copy number [216]. Such a reduction points to compromised mitochondrial biogenesis, further supported by the downregulation of genes associated with mitochondrial replication and repair [216]. Collectively, these findings highlight that excessive fructose consumption may contribute to both hepatic and testicular dysfunction by impairing mitochondrial integrity and altering key metabolic pathways, thereby increasing the risk of developing metabolic diseases [217] and infertility [47, 197].

## 16. An Integrated Model of Obesity: Exploring the Roles of Energy Balance, Carbohydrate‐Insulin, Oxidation–Reduction, and Obesogens Models

Obesity is commonly associated with excessive calorie intake, a concept central to the energy balance model (EBM). According to this model, obesity arises from an imbalance between energy intake and expenditure [109]. Specifically, weight gain occurs when caloric consumption exceeds the body’s daily energy requirements. The EBM posits that the brain regulates body weight by integrating internal and external signals through multiple mediators. Thus, disruptions in these regulatory signals may lead to overeating and obesity. Central to this model is the role of food intake regulation, with particular emphasis on the contribution of UPFs, which are known to stimulate overconsumption, increase adiposity, impair insulin sensitivity, and promote weight gain [218]. Despite its widespread acceptance, the EBM has notable limitations.

It fails to account for the paradoxical rise in obesity rates after 2000, despite reported reductions in caloric intake and increases in physical activity levels [219]. Furthermore, it cannot explain why individuals in 2006 had higher BMI values than those in 1988 despite similar caloric intake and physical activity levels [220]. Additionally, the EBM does not adequately incorporate the influence of early‐life nutrition, developmental programing, or environmental exposures on long‐term obesity risk [110].

In contrast, the carbohydrate‐insulin model (CIM) offers a different perspective by focusing on hormonal regulation and energy partitioning [221]. This model suggests that refined carbohydrates trigger insulin spikes, which, in turn, promote fat storage. According to the CIM, fast‐digesting carbohydrates increase insulin levels, leading to the activation of LPL, inhibition of the adrenergic system, and suppression of lipolysis in adipose tissue, thereby favoring energy storage over expenditure [221]. Unlike the EBM, which emphasizes calorie quantity and neural regulation, the CIM highlights the metabolic and endocrine consequences of carbohydrate quality and glycemic load [222]. It further proposes that high‐glycemic carbohydrates foster insulin resistance and dysregulate hypothalamic signaling pathways that control hunger and energy homeostasis [223]. However, the CIM also has explanatory gaps. It does not account for the rising prevalence of obesity in infants whose diets are primarily milk‐based [224] nor does it adequately address the role of epigenetic mechanisms and transgenerational inheritance in the pathogenesis of obesity [225].

The obesogen model presents an environmentally focused framework that links obesity to EDCs, known as obesogens, which increase white adipose tissue (WAT) mass in vivo [226]. Potential obesogens can promote adipocyte differentiation in vitro but have not yet been confirmed to increase WAT accumulation in living organisms. These chemicals influence adipose tissue by altering stem cell commitment, enhancing adipocyte formation, and modifying fat cell number, size, and TG content [227]. They also interfere with hormonal signaling pathways, particularly those involving peroxisome proliferator‐activated receptors (PPARs), retinoid X receptors (RXRs), and thyroid hormone receptors [225]. Disruptions of these pathways affect adipogenesis, lipid metabolism, and long‐term metabolic programing [225]. Obesogens exert effects across multiple organs, including adipose tissue, the liver [228, 229], the pancreas [230, 231], the gastrointestinal tract, the brain [225, 232], and reproductive organs [233–235], thereby altering the energy balance and increasing vulnerability to obesity.

These compounds are prevalent in common consumer products, such as food packaging, plastics, pesticides, personal care products, and industrial pollutants [110, 225]. Notably, exposure during sensitive developmental windows, such as in utero or during early postnatal life, can epigenetically reprogram the metabolic “set points” toward increased fat accumulation, with effects that may persist across generations [110, 225]. Heindel et al. [110] proposed that the global obesity epidemic is partially driven by environmental exposure to obesogenic chemicals, many of which are abundant in UPFs. These chemicals interfere with normal metabolic development by disrupting endocrine signaling and altering ROS homeostasis, thereby contributing to oxidative stress and metabolic dysfunction. Consequently, the authors advocate for integrating the OBS/REDOX model, which suggests that environmental chemicals (found in air, food, food packaging, and household products) produce misleading autocrine and endocrine metabolic signals, including ROS. These signals undermine normal energy‐regulation processes, elevate both basal and stimulated insulin secretion, reduce energy efficiency, and affect appetite and energy expenditure, ultimately contributing to weight gain [110, 225]. Integrating this perspective highlights the importance of obesogens and redox imbalance within existing frameworks such as the EBM and CIM, offering a more complete understanding of obesity’s underlying causes [110].

**Figure 6:**
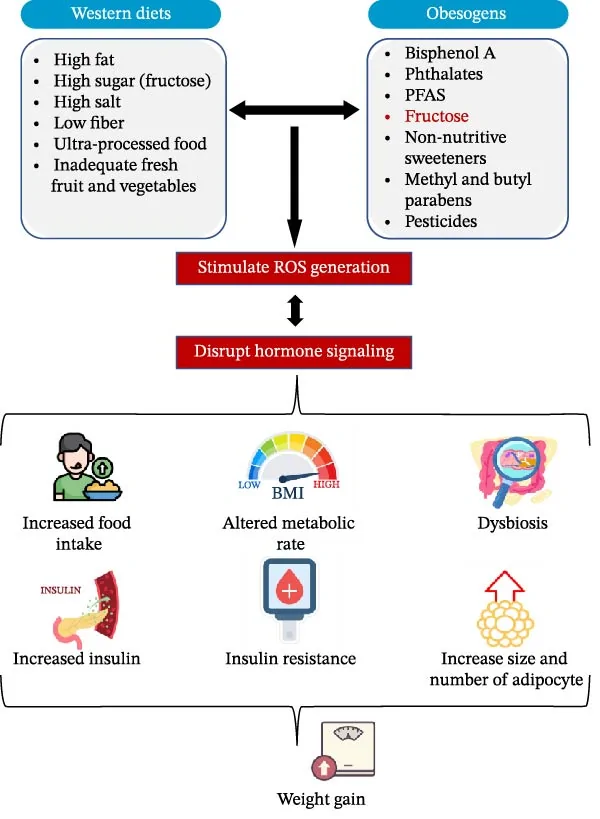
The OBS/REDOX model offers a comprehensive theory to explain the global increase in obesity. Exposure to obesogens during pregnancy and throughout life triggers incorrect hormonal and ROS signaling, which disrupts metabolism. The outcomes influenced by the OBS/REDOX model align with those suggested by the EBM and CIM models. Therefore, the OBS/REDOX model synthesizes all existing models, providing a cohesive framework for understanding the mechanisms behind the obesity epidemic.

This integrated model underscores how chronic exposure to obesogens alters gene expression profiles in key metabolic tissues, partly through epigenetic and oxidative stress‐related mechanisms [236]. Figure 6 illustrates the interactions among dietary factors, obesogens, ROS, and obesity outcomes. According to the OBS/REDOX model, adopting a low‐carbohydrate diet or reducing UPF consumption may ameliorate ROS‐mediated metabolic disruptions by lowering both obesogen exposure and oxidative stress.

The divergence between the EBM and CIM reflects a broader debate concerning the relative importance of macronutrient quantity versus quality in the pathogenesis of obesity. The OBS/REDOX model extends this discussion by emphasizing the obesogenic burden of UPFs, particularly through the lens of redox biology. UPFs, rich in obesogenic compounds, have been linked to heightened inflammation, mitochondrial dysfunction, enhanced insulin secretion, TG accumulation, and reduced insulin sensitivity [237]. Together, these models illustrate the multifactorial nature of obesity and highlight the need for an integrative approach that encompasses dietary composition, hormonal regulation, environmental exposures, and redox dynamics.

Excess nutrient intake and exposure to dietary obesogens have been implicated in the development of hyperinsulinemia, a condition that further promotes adipose tissue expansion and metabolic dysregulation [238]. Insulin plays a crucial role in fat storage by enhancing glucose uptake and stimulating lipogenesis in adipocytes [238]. Notably, ROS have been shown to directly stimulate insulin secretion even in the absence of glucose [108, 239], highlighting their role as metabolic signaling molecules. Conversely, the elimination of ROS has been associated with a reduction in insulin secretion, underscoring their importance in regulating insulin secretion [240].

The OBS/REDOX model offers a mechanistic framework to explain the observed clinical benefits of dietary interventions that reduce UPF consumption and carbohydrate intake, thereby aligning with the principles of the CIM [241]. This model underscores the importance of minimizing obesogen exposure, particularly during critical developmental windows such as pregnancy, infancy, and early childhood, when metabolic programing is most susceptible to disruption. Practical recommendations derived from this model include prioritizing organic foods, substantially reducing UPF consumption, avoiding plastic containers, using fragrance‐free personal care products, replacing nonstick cookware with safer alternatives, and consuming purified drinking water. In addition to individual‐level behavioral changes, the model emphasizes the urgent need for public health regulations and policy interventions aimed at limiting the pervasive presence of obesogenic chemicals in consumer products and the environment [110].

In brief, emerging research across various obesity models, including the EBM, CIM, and the OBS/REDOX model, collectively underscores the critical role of fructose, particularly from UPFs, in promoting metabolic dysfunction. While the EBM emphasizes caloric imbalance and the CIM highlights insulinogenic responses to refined carbohydrates, recent insights from the OBS/REDOX model reveal that excessive fructose intake not only drives hyperinsulinemia and adipose tissue expansion but also acts as a dietary obesogen by increasing ROS and altering mitochondrial function. Fructose‐induced oxidative stress has been shown to exacerbate insulin secretion, inflammation, lipogenesis, and insulin resistance, all core features of the MetS and obesity. Moreover, chronic fructose consumption may contribute to long‐term metabolic reprograming through redox‐sensitive and epigenetic pathways, especially when exposure occurs during early development. These findings reinforce the need to limit dietary fructose, primarily through the reduction of UPFs, and support broader regulatory measures to minimize obesogen exposure as part of an integrative strategy to address the obesity epidemic.

## 17. At What Point do Obesity and Male Infertility Intersect?

The detrimental effects of obesity on sperm quality and hormone regulation are well established; however, robust population‐based epidemiological evidence has mainly emerged during recent decades [242].

In 2006, Sallmen et al. [243] conducted a large‐scale study involving 20,620 families from Iowa and North Carolina. Their findings demonstrated a dose–response association between BMI and male fertility, showing that male fertility decreased with every 3‐point increase in BMI above 25 kg/m^2^, with an odds ratio (OR) of 1.12 (95% confidence interval [CI] 1.01–1.25). These results have since been corroborated by multiple international studies conducted in Denmark [244], Norway [245], North America [246], and France [247], which consistently support a negative relationship between obesity and male reproductive function.

Spermatogenesis is critically dependent on adequate intratesticular testosterone concentrations [248–250], and the regulation of male reproduction is primarily centered on the hypothalamic–pituitary–gonadal (HPG) axis. However, the evidence suggests that several additional metabolic and reproductive hormones, such as leptin [251–253] and kisspeptin [254, 255], may also modulate this axis.

A decline in circulating testosterone may be accompanied by reduced intratesticular testosterone production. The HPG axis operates via the pulsatile release of gonadotropin‐releasing hormone (GnRH) from the hypothalamus, stimulating the anterior pituitary to LH and FSH. The LH stimulates Leydig cells to produce testosterone, which binds to sex hormone‐binding globulin (SHBG) or is aromatized to estradiol by the enzyme aromatase [256, 257]. Although FSH alone is not sufficient to maintain complete spermatogenesis, it acts synergistically to optimize SC function within the seminiferous tubules [256, 258]. In the context of obesity, hypogonadism is often characterized by impaired LH pulsatility and reduced testicular responsiveness to LH stimulation [259]. Elevated levels of leptin and insulin, along with increased pro‐inflammatory cytokines, can disrupt normal GnRH pulsatility, thereby altering downstream LH and FSH secretion [259–261]. Additionally, the Leydig cells may also exhibit reduced sensitivity to LH due to changes in their receptor expression or signaling pathways, which can be influenced by factors such as increased estrogen levels resulting from enhanced aromatase activity in the adipose tissue [262, 263]. This hormonal dysregulation may exacerbate testosterone deficiency and contributes to reproductive dysfunction.

Furthermore, obese men experience an increased aromatization of testosterone to estradiol (E2) and estrone, which may suppress hypothalamic kisspeptin signaling and further reduce HPG axis activity [263]. Adipose tissue‐derived leptin, which is crucial for energy homeostasis, is often elevated in obesity, resulting in central leptin resistance [264]. Although mutations in the leptin gene or receptor can lead to overconsumption and decreased energy expenditure, obese individuals often have chronically high leptin levels accompanied by reduced leptin responsiveness [265, 266]. This hypothalamic resistance reduces leptin’s ability to stimulate the HPG axis, contributing to lower testosterone levels [265]. Collectively, these endocrine disruptions may reduce spermatogenic efficiency and alter the epigenetic programing of sperm [267].

Research suggests that disruption of the sperm epigenome may affect normal gamete function and potentially influence subsequent embryonic development [268]. Since sperm serve two key functions—delivering the paternal genome to the oocyte and contributing molecular factors involved in embryogenesis and offspring development [269]—it is crucial to evaluate how epigenetic changes impact both roles. Recent studies have shown that sperm retain specific epigenetic signatures—including DNA methylation, histone retention and modification, protamine content, and non‐coding RNAs—that may contribute to early development [270–272]. These epigenetic features are highly sensitive to environmental influences such as diet, BMI, physical activity, aging, and exposure to environmental toxins or EDCs [273–277].

McPherson et al. [278] demonstrated that obesity‐related comorbidities—including systemic inflammation, glucose intolerance, psychological stress, and hypercholesterolemia—are strong predictors of altered sperm microRNA (miRNA) profiles and may influence offspring metabolic phenotypes. Notably, preconception dietary and exercise interventions in obese males were found to partially normalize these miRNA signatures, thereby improving paternal metabolic health and metabolic outcomes in female offspring. There is also a well‐documented association between aberrant sperm DNA methylation and declines in motility, morphology, and DNA integrity [279–282].

In summary, available evidence suggests that obesity‐induced molecular alterations within sperm can compromise fertility and may influence offspring health. Accumulating evidence indicates that the epigenomic footprint of male overweight and obesity is detectable in sperm [267, 283, 284], and these modifications have the potential to influence the health of subsequent generations [285–287]. Importantly, many of these changes appear to be at least partially modifiable, underscoring the importance of early lifestyle interventions and environmental risk mitigation strategies for supporting male reproductive health.

## 18. Paternal Aging as a Modifier of Male Reproductive Health

Unlike the relatively abrupt loss of female reproductive capacity at menopause, male reproductive aging is generally gradual and does not have a universally accepted clinical threshold. Nevertheless, age‐related changes may become detectable from the mid‐30 s onward and tend to become more evident in later decades. Current evidence most consistently associates advancing paternal age with reduced semen volume and sperm motility and increased sperm DNA fragmentation, whereas findings for SC, TSC, and morphology remain more heterogeneous. A systematic review found that semen volume declined in seven of nine recent studies, motility declined in 9 of 10 studies, and DNA fragmentation increased in all 11 studies that assessed this outcome [288]. Although the reported effect sizes were generally modest, their cumulative impact may prolong the time to pregnancy and reduce reproductive success. Proposed mechanisms include age‐related mitochondrial dysfunction, increased ROS production, impaired chromatin packaging, and reduced DNA repair capacity, which may increase the susceptibility of sperm DNA to oxidative injury [288].

Paternal age may also interact with metabolic dysfunction rather than acting as an isolated determinant of reproductive aging. The prevalence of obesity, insulin resistance, diabetes, hypertension, dyslipidemia, and hypogonadism increases with age, and these conditions may further impair hormonal regulation, testicular function, redox balance, and sperm quality [288]. Accordingly, aging may increase susceptibility to the reproductive consequences of chronic metabolic stress, although direct evidence demonstrating a specific interaction between paternal age and dietary fructose remains limited. Importantly, the meta‐regression analyses by Levine et al. included age among the predetermined covariates, and the temporal declines in SC and TSC remained significant after age adjustment and related sensitivity analyses [1, 34]. Thus, population aging alone is unlikely to explain the reported decline in sperm counts; instead, paternal age should be regarded as an independent and potentially modifying factor within the broader interaction among dietary exposure, metabolic dysfunction, and male reproductive health.

## 19. Conclusions and Future Directions

The relationship between obesity and male fertility has garnered increasing attention as a critical public health issue. Research has identified an association between rising obesity rates and declining male reproductive health, with dietary factors, particularly excessive fructose intake, representing potential contributors. Fructose consumption, primarily through HFCS and processed foods, has been linked to MetS, hormonal disruption, and impaired sperm function. Unlike glucose metabolism, hepatic fructose metabolism bypasses key regulatory steps of glycolysis and may promote ATP depletion, uric acid production, de novo lipogenesis, insulin resistance, and oxidative stress. These disturbances contribute to obesity, hyperglycemia, dyslipidemia, and hypertension, which are major features of MetS, which negatively affects general health and specifically male reproductive function.

Experimental studies suggest that high‐fructose diets may reduce testosterone levels, increase sperm DNA damage, and promote oxidative stress, a mechanism widely implicated in male infertility. Experimental models further indicate that prolonged fructose intake leads to structural changes in the testes, increases germ cell apoptosis, and impairs spermatogenesis. Testicular insulin signaling, which is important for normal reproductive development and function, is also disrupted by fructose‐induced insulin resistance, further compromising testicular and sperm function.

In addition to fructose, other obesogenic chemicals, including EDCs, may exacerbate metabolic and reproductive dysfunction. These chemicals can interfere with signaling pathways involving PPARs and other nuclear receptors, thereby promoting adipogenesis and potentially inducing long‐term metabolic alterations. Combined exposure to obesogenic chemicals and high‐fructose diets may reinforce metabolic dysfunction, hormonal imbalance, and impaired reproductive function. Furthermore, chronic fructose consumption may disrupt hunger and satiety mechanisms by reducing leptin sensitivity and impairing the regulation of food intake. This dysregulation may promote overeating, weight gain, and persistent metabolic stress. Chronic activation of the proposed survival pathway, which may have evolved to promote energy storage during periods of food scarcity, could therefore contribute to the current obesity epidemic. The resulting energy surplus may intensify hormonal imbalances and further exacerbate metabolic and reproductive disorders.

Obesity itself is a multifactorial disease, and despite decades of research, a single etiological factor is yet to emerge. Altered nutrition during pregnancy is an important risk factor for postnatal weight gain, largely through developmental programing, but this aspect is not fully incorporated into existing obesity models like the CIM or EBM. By contrast, the OBS/REDOX model emphasizes the effects of developmental exposure to obesogenic chemicals, which may generate inappropriate autocrine and endocrine signals in metabolic tissues and increase susceptibility to weight gain later in life and potentially across generations. This testable OBS/REDOX model aligns with aspects of both the EBM and CIM proposes that misleading cellular signals can stimulate food consumption, insulin release, and fat storage, even when energy intake is not excessive. This framework provides valuable insights into changes in food intake and alterations in energy efficiency and storage.

In response to these growing concerns, public health strategies may include measures to reduce excessive intake of added fructose, particularly from HFCS‐containing beverages and UPFs. Policymakers could consider appropriate regulatory and labeling measures, while public awareness campaigns can educate the public about the risks associated with high‐fructose diets. Nutrition counseling and metabolic interventions play crucial roles in reversing obesity‐related infertility. Targeted therapeutic strategies, such as antioxidant supplementation, may help mitigate fructose‐induced oxidative stress. Lifestyle interventions, including increased physical activity and adherence to a Mediterranean‐style diet, have been shown to restore the hormonal balance and improve sperm parameters. Furthermore, research into dietary modifications and fertility treatments should consider the impacts of fructose metabolism on reproductive hormones, and further investigation into obesogens and endocrine disruptors is necessary to assess their long‐term effects on fertility rates. Ultimately, understanding the complex interactions between diet, metabolism, and reproductive health is essential for developing effective strategies to combat obesity‐related infertility and improve public health.

## Author Contributions

**Mohammad Hossein Nasr-Esfahani:** conceptualization, writing – original draft, writing – review and editing. **Marziyeh Tavalaee**: conceptualization, writing – original draft, writing – review and editing. **Nushin Naderi**: visualization, writing – review and editing. **Mazdak Razi**: writing – review and editing.

## Funding

No funding was received for this manuscript.

## Disclosure

All the authors confirmed the final version.

## Conflicts of Interest

The authors declare no conflicts of interest.

## Data Availability

Data sharing is not applicable to this article as no datasets were generated or analyzed during the current study.
